# Physicochemical Characterization and Functional Analysis of the Polysaccharide from the Edible Microalga *Nostoc sphaeroides*

**DOI:** 10.3390/molecules23020508

**Published:** 2018-02-24

**Authors:** Haifeng Li, Linnan Su, Sheng Chen, Libin Zhao, Hongyu Wang, Fei Ding, Hong Chen, Ruona Shi, Yulan Wang, Zebo Huang

**Affiliations:** 1 Center for Bioresources & Drug Discovery and School of Biosciences & Biopharmaceutics, Guangdong Pharmaceutical University, Guangzhou 510006, China; lihf@gdpu.edu.cn (H.L.); sulinnan1234@foxmail.com (L.S.); 15626200071@163.com (H.W.); 15692435199@163.com (F.D.); hongchen1117@163.com (H.C.); 15521217084@163.com (R.S.); 2 Research & Development Centre, Hunan Yandi Bioengineering Co., Ltd., Zhuzhou 412000, China; chensheng@ydhn.com (S.C.); zhaolibin@ydhn.com (L.Z.); wangyulan@ydhn.com (Y.W.); 3 School of Food Science and Engineering, South China University of Technology, Guangzhou 510641, China

**Keywords:** *Nostoc sphaeroides*, polysaccharide, *Caenorhabditis elegans*, oxidative stress, apoptosis

## Abstract

*Nostoc* colonies have been used as food and medicine for centuries, and their main supporting matrix is polysaccharides, which help *Nostoc* cells resist various environmental stresses including oxidative stress. Here we isolated a polysaccharide, nostoglycan, from cultured *Nostoc* *sphaeroides* colonies and determined its physicochemical properties, which revealed a characteristic infrared absorption spectrum typical of polysaccharides and an amorphous morphology with rough surfaces. We also show that nostoglycan has strong moisture absorption and retention capacities and a high relative viscosity. Using *Caenorhabditis elegans* models, we then demonstrate that nostoglycan is capable of improving overall survival rate of the animals under increased oxidative stress caused by paraquat. Nostoglycan also reduces reactive oxygen species level, inhibits protein carbonyl formation and lipid peroxidation, and increases activities of superoxide dismutase and catalase in paraquat-exposed nematodes. As oxidative stress may drive tumor progression, we further demonstrate that nostoglycan can suppress the proliferation of several types of tumor cells and induce apoptosis of human lung adenocarcinoma A549 cells via caspase-3 activation. Together, our results yield important information on the physicochemical characteristics and demonstrate the antioxidant and anti-proliferative functions of nostoglycan, and thus provide an insight into its potential in food and health industries.

## 1. Introduction

*Nostoc* is a genus of nitrogen-fixing cyanobacteria that can form gelatinous colonies composed of filaments of moniliform cells in a range of environments. *Nostoc sphaeroides*, for instance, grows naturally in the form of spherical macrocolonies of dark green appearance during the winter in some mountain paddy fields in China [1]. Traditionally, *N. sphaeroides* and other *Nostoc* colonies have been collected from natural habitats and used as food delicacy and herbal medicine in Asia, Europe, and America [1,2,3,4]. For example, *N. sphaeroides* is known as Ge-Xian-Mi in Chinese, named after Ge Hong, an influential scholar in Taoism, alchemy and medicine in Jin Dynasty (265–420). According to the autobiography of the last Chinese emperor Henry Pu Yi, Ge-Xian-Mi was fried with diced duck meat and served at royal meals of Qing Dynasty (1644–1912). In *Supplement to Compendium of Materia Medica* (*Bencao Gangmu Shiyi*) written by Zhao Xuemin (1719–1805) in Qing Dynasty, *N. sphaeroides* was recorded to treat a variety of medical conditions such as inflammation, burns, hypertension, nyctalopia and chronic fatigue. Interestingly, recent studies have revealed the nutritional and pharmacological benefits of *N. sphaeroides* (or *Nostoc commune* var. *sphaeroides*), including anti-inflammatory, anti-hyperlipidemic, and atheroprotective effects [5,6,7].

Endogenous reactive oxygen species (ROS) are byproducts of normal metabolism of oxygen, primarily generated from the respiratory chain of mitochondria [8]. These small reactive molecules are normally present at low levels inside cells and play important roles in a range of biological processes such as regulation of cell signaling and homeostasis [9]. Due to their highly reactive nature, however, excessive ROS can make cellular macromolecules vulnerable to oxidation [8]. Under persistent intrinsic as well as extrinsic stress, ROS levels may increase to an extent that exceeds physiological levels and overwhelms the antioxidant defense machinery of cells. When ROS production outpaces ROS scavenging by the endogenous antioxidant system, an excessive accumulation of ROS occurs and induces a state of oxidative stress, which may cause damage to cellular components and result in a disruption of cellular function [8]. It is also established that oxidative stress is implicated in a number of diseases and conditions, including cancer, inflammation, and neurodegeneration [10,11]. A high ROS level, for instance, can trigger carcinogenesis and promote tumor progression via cell proliferation and cell death pathways [10]. Therefore, amelioration of oxidative stress and associated damages represents a valuable approach for health maintenance and disease prevention. For example, adequate consumption of food-derived antioxidants such as antioxidant polysaccharides has been reported to reduce oxidative damages and to be beneficial to both human and animal health [12,13,14].

*Nostoc* colonies are commonly grown on soil surface and thus have developed a number of strategies to resist various environmental stresses, including extreme desiccation and intense solar ultraviolet radiation, both of which can stimulate ROS generation and induce oxidative damages [1,15,16]. The major adaptive strategies of *Nostoc* against desiccation, ultraviolet radiation, and other environmental conditions are closely associated with their abundant extracellular polysaccharides [1,4,15]. Interestingly, these polysaccharides are also shown to have beneficial effects such as complement-fixing and antioxidant activities [2,4,17]. For example, a polysaccharide from *N. sphaeroides* is capable of scavenging superoxide, hydroxyl, and DPPH radicals, demonstrating its in vitro antioxidant capacity [17]. However, in vivo antioxidant studies as well as physicochemical characterization of *N. sphaeroides* polysaccharides are lacking. Here we first determined the physicochemical properties of a polysaccharide isolated from *N. sphaeroides* (nostoglycan). Then we tested the in vivo antioxidant capacities of nostoglycan using *Caenorhabditis elegans*, a simple but powerful animal model widely used in biomedical studies, under paraquat-induced oxidative stress. We further examined the anti-proliferative activities of nostoglycan using several human tumor cell lines as oxidative stress is known to be involved in cancer development. Finally, we investigated the effect of nostoglycan on cell apoptosis and caspase-3 activation.

## 2. Results and Discussion

### 2.1. Physicochemical Characterization of Nostoglycan

The yield of water-soluble polysaccharide from *N. sphaeroides* (nostoglycan) was ~30% after removal of proteins by papain digestion and purification by ethanol precipitation, and its total carbohydrate content was ~80%. The molecular weight distribution of nostoglycan was assessed by high-performance gel permeation chromatography [18], which showed a distinct peak (data not shown) with a weight-average molecular weight (Mw) of 1.99 × 10^3^ kDa and a number-average molecular weight (Mn) of 0.99 × 10^3^ kDa. The monosaccharide molar ratio of nostoglycan was 34.5% mannose, 21.8% fructose, 14.6% galactose, 17.7% glucose, 6.1% xylose, 2.2% rhamnose, and 3.1% galacturonic acid. As revealed previously using methylation and gas chromatography-mass spectrometry analysis, xylose, glucose, and galactose were 1→4 linked; mannose, galactose, and xylose were terminal groups; and branch points occurred in glucose as 1→3,4 and 1→3,6 linkages and in xylose as a 1→3,4 linkage [1].

**Figure 1:**
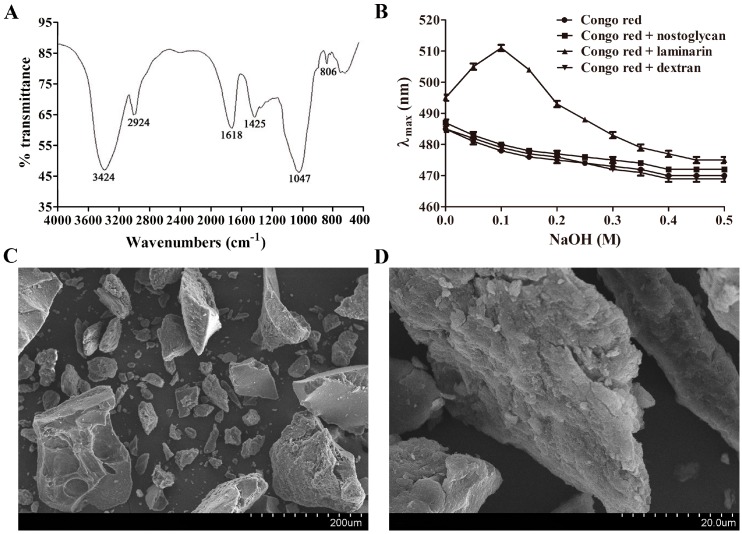
Spectroscopic and physicochemical characterization of nostoglycan. (**A**) FTIR spectrum of nostoglycan in the frequency range of 4000–400 cm^−1^; (**B**) Triple helical conformation analysis of polysaccharides in aqueous solution. The maximum absorption wavelength of Congo red in the absence or presence of an indicated polysaccharide was recorded by a spectrophotometer under increasing alkaline conditions (0–0.5 M NaOH); (**C**,**D**) Surface morphology of nostoglycan by scanning electron microscopy at 200× and 2000× magnification, respectively.

Fourier transform infrared (FTIR) spectroscopy is a powerful tool to identify major organofunctional groups and chemical bonds in polymers, and thus was used to characterize the spectroscopic properties of nostoglycan. As shown in Figure 1A, a broad and intense peak at 3424 cm^−1^ was observed, demonstrating the presence of the stretching vibration of hydroxyl groups. The bands at 2924 cm^−1^ and 1425 cm^−1^, which can be attributed to C–H stretching vibration and deforming vibration, respectively, were also present in the spectrum. The peaks at 1618 cm^−1^ and 1047 cm^−1^ were assigned to the stretching vibrations of C=O and C–O, respectively, indicating the existence of carboxyl groups. Moreover, the band at 806 cm^−1^ was consistent with the presence of α-type glycosidic linkages between glycosyl residues. Collectively, the infrared spectrum of nostoglycan revealed the characteristic absorption bands typical of polysaccharides.

Polysaccharides in aqueous solution can have different chain conformations such as random coil, various helices, aggregate, and sphere [19]. Interestingly, the bioactivities of some polysaccharides may, as reported, be related to their triple helical conformation [19,20,21]. For example, lentinan, an antitumor β-1,3-d-glucan isolated from the mushroom *Lentinus edodes*, is shown to have a triple helical conformation [19]. Therefore, we tested whether nostoglycan can form a triple helical conformation in aqueous solution using Congo red binding assay, a valuable approach that has been used to detect conformational transition of triple helix to coil in various polysaccharides, including heteropolysaccharides [21]. The anionic dye Congo red can combine polymers with triple helical structure, resulting in a red shift of the maximum absorption [20]. However, alkali such as NaOH can disrupt intermolecular as well as intramolecular hydrogen bonds in triple helix, inducing a conformational transition to random coil [20]. As shown in Figure 1B, the maximum absorption wavelength (λ_max_) of Congo red and laminarin mixture first increased from 495 nm to 511 nm as NaOH concentration increased to 0.1 M and then gradually decreased when NaOH concentration increased further to 0.5 M, indicating the formation of a Congo red-laminarin complex. However, the bathochromic shift was not observed when Congo red was incubated with nostoglycan or dextran, suggesting the absence of a complex with Congo red. These results indicate that nostoglycan does not form a triple helical conformation in aqueous solution.

The surface morphology of substances is closely associated with their physicochemical properties and may affect their biological functions [22]. Scanning electron microscopy (SEM) is known to be an efficient qualitative tool for characterization of microstructural properties such as roughness, porosity, crystallinity, and orientation, and was thus used to investigate the surface morphology of nostoglycan. As shown in Figure 1C, nostoglycan was present as irregular and angular particles with rough surfaces under SEM at 200× magnification, indicating an amorphous nature of the polysaccharide. A higher magnification at 2000× revealed the multilayer and flaky morphologies of nostoglycan (Figure 1D). Interestingly, the polysaccharide produced by *Nostoc flagelliforme*, another edible terrestrial *Nostoc* species grown in arid and semi-arid areas, is also shown to be porous aggregates with a scaly surface under SEM [23]. However, the surface morphology of polysaccharides may be affected by a number of factors, including preparation methods. For example, spray-dried grewia polysaccharide gum has a more spherical shape and smoother surface as compared to air-dried and freeze-dried grewia polysaccharides [24]. Therefore, further investigation is needed to provide more details on the morphological features of nostoglycan and other *Nostoc* polysaccharides.

**Figure 2:**
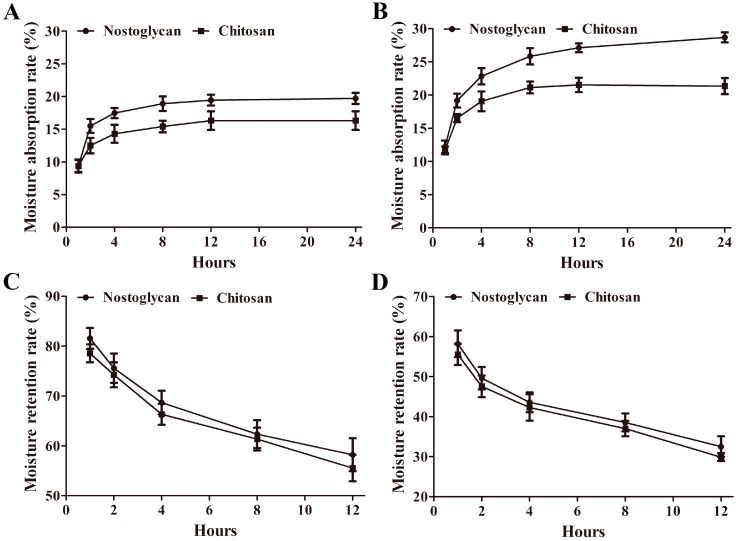
Moisture absorption and retention rates of nostoglycan. For moisture absorption analysis, the dried polysaccharides were placed at 43% (**A**) or 81% (**B**) RH for the indicated times. For moisture retention determination, the humidified polysaccharides were first placed at 43% RH for 12 h (**C**) and then in a silica gel chamber for the indicated times (**D**). Both moisture gain and loss were determined gravimetrically. Data are representative of three independent experiments and shown as mean ± SD.

The industrial applications of polysaccharides are often associated with their physical characteristics, such as moisture property and viscosity. Interestingly, the extracellular polysaccharide matrix is also considered to play a critical role in the morphology maintenance of *Nostoc* colonies per se, particularly during desiccation and rewetting processes [1,25]. We have previously analyzed the moisture buffering capacities of polysaccharide from *N. commune* colonies and the viscosities of polysaccharides from field colonies and suspension cultures of three edible *Nostoc* species [1,4]. Here we investigated the moisture absorption and retention capabilities of nostoglycan using weight gain-and-loss assay. When the polysaccharides were placed at 43% relative humidity (RH), the moisture absorption rate (*R*a) of nostoglycan was increased at a faster pace than chitosan in the first 12 h and the *R*a of nostoglycan and chitosan was 19.7% and 16.3%, respectively, after 24 h (Figure 2A). When the samples were exposed to 81% RH, the *R*a of both nostoglycan and chitosan was higher than that at 43% RH and reached 28.7% and 21.4%, respectively, after 24 h (Figure 2B). To assess the moisture-retention capacity, the polysaccharides were first placed in a water-humidified chamber for 24 h and then dehydrated at 43% RH. As shown in Figure 2C, the moisture retention rate (*R*r) of both nostoglycan and chitosan was gradually decreased upon dehydration at 43% RH, which was 58.2% and 55.6%, respectively, after 12 h. When the samples were further dehydrated in a silica gel chamber, the water loss of nostoglycan and chitosan in the complete dry condition was, as expected, much faster than at 43% RH and the *R*r was decreased to 32.5% and 29.9%, respectively (Figure 2D). Together, these results demonstrate the strong moisture absorption and retention capacities of nostoglycan, which is comparable with chitosan and in agreement with the polysaccharide from *N. commune* [4]. The rapid water uptake and slow water loss of *Nostoc* polysaccharides may help *Nostoc* colonies to maintain their morphology and survive at varying humidity conditions. For example, when the desiccated colonies of *N. commune* were rehydrated, the brittle polysaccharide matrix can rapidly swell into a loose and porous network, which allows fast water absorption [26,27]. On the other hand, this stereoscopic network structure can also efficiently prevent loss of water from the colonies during desiccation [27].

Rheological properties of polysaccharides may also affect their water retention capacity and application potentials. For example, hyaluronic acid, a polysaccharide naturally present in the extracellular matrix of vertebrate tissues, has been widely used as moisturizer and humectant in a range of biomedical and cosmetic products due, at least partly, to its high viscosity. Therefore, we measured the viscosity of nostoglycan, which showed a high relative viscosity (1.78) as compared to pectin and agar (1.56 and 1.39, respectively). This is in agreement with our previous findings that the polysaccharides isolated from *Nostoc* colonies collected from the field also have high kinematic viscosities [1]. A recent study has revealed that *N. sphaeroides* polysaccharides can form a gel system in aqueous solution, which may be associated with the branch structures of the polysaccharides [28]. Interestingly, the polysaccharides from *N. commune* are shown to contain the nosturonic acid {3-*O*-[(*R*)-1-carboxyethyl]-d-glucuronic acid} pendant group, which is proposed to play a role in modulating the specific rheological property of *Nostoc* polysaccharides and in the water flux of *Nostoc* colonies [25,26]. To date, however, the presence of nosturonic acid in the polysaccharides of *N. sphaeroides* remains unknown. Together, these results suggest that the viscous *Nostoc* polysaccharides not only have a moisture-buffering effect in the maintenance of their colony morphology but also have industrial potentials as thickening and emulsifying agents.

### 2.2. Increase of Survival Rate and Reduction of ROS Levels by Nostoglycan in C. elegans under Oxidative Stress

**Figure 3:**
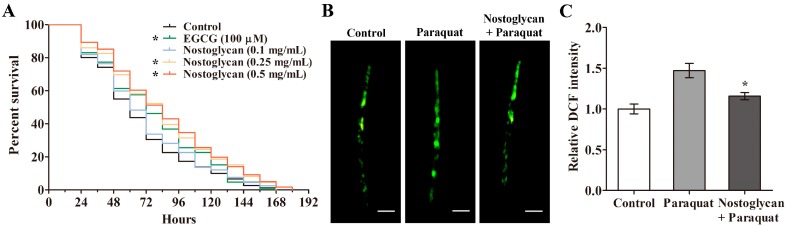
Effect of nostoglycan on the survival rate and ROS level of *C. elegans* under oxidative stress. (**A**) Oxidative survival curves of nematodes with or without nostoglycan treatment. Young adult nematodes were first treated with nostoglycan or EGCG at the indicated concentrations and then exposed to 70 mM paraquat. Live nematodes were scored every 12 h until all dead. Representative Kaplan–Meier curves are presented from three independent experiments; (**B**) Representative micrographs of DCF fluorescence in paraquat-exposed nematodes. The nematodes were treated with or without 0.5 mg/mL nostoglycan prior to 10 mM paraquat exposure and then stained with the fluorescent probe DCFH-DA. The fluorescent images were captured by an ImageXpress Micro System. Scale bars, 200 μm; (**C**) DCF fluorescence intensity of paraquat-exposed nematodes. The nematodes were treated as in (**B**), and the DCF intensity was measured with a fluorescence microplate reader after DCFH-DA treatment. Data are presented as mean ± SEM of three independent experiments. * p < 0.05 (as compared to paraquat-exposed nematodes).

Extensive studies suggest that persistent oxidative stress can accelerate ageing process and reduce life expectancy in animal models, and dietary supplementation of antioxidants is shown to be a promising strategy to reduce oxidative damages and increase oxidative survival [29,30,31,32]. The polysaccharides isolated from *N. sphaeroides* have been previously shown to scavenge free radicals in vitro [17], suggesting their potential to attenuate free radical-mediated oxidative stress. An in vitro antioxidant effect, however, does not necessarily imply an in vivo antioxidant function. Thus, we investigated the antioxidant activity of nostoglycan in *C. elegans*, a convenient animal model for stress survival studies, under increased oxidative stress elicited by paraquat, which is a potent ROS generator known to induce oxidative damages in animals and humans [33]. Wild-type nematodes were first treated with 0–0.5 mg/mL of nostoglycan for 48 h and then exposed to 70 mM paraquat, and the survival rates were determined. As shown in Figure 3A, the survival rates of nematodes pretreated with nostoglycan at 0.25 and 0.5 mg/mL were increased as compared to that of the nematodes exposed to paraquat alone (*p* < 0.05), at a similar level with the well-known antioxidant epigallocatechin-3-gallate (EGCG). These results indicate that nostoglycan is capable of attenuating oxidative damages in animal models.

An excessive accumulation of ROS is known to be a major cause of oxidative stress [8], while prevention of ROS overproduction represents an efficient way to restore cellular redox balance and thus alleviate oxidative impairments. For example, a polysaccharide isolated from the mushroom *Dictyophora indusiata* is recently shown to attenuate chemosensory behavior dysfunction through reducing ROS level in transgenic *C. elegans* models of neurodegeneration, where expression of disease proteins induces ROS overproduction and causes neuronal damages [29]. Since paraquat can induce endogenous ROS overproduction, we investigated whether the protective effect of nostoglycan against oxidative stress induced by paraquat was through modulation of ROS level. As shown above, 70 mM paraquat induced nematode death in a relatively short time (<24 h), and therefore, in order to make ROS determinations, we used a lower dose of paraquat (10 mM) to induce oxidative stress but also to ensure the nematodes were still alive. The nematodes were first treated with 0.5 mg/mL nostoglycan for 48 h and then exposed to 10 mM paraquat for 24 h, and the ROS level was determined using 2′,7′-dichlorodihydrofluorescein diacetate (DCFH-DA) fluorescent probe. As shown in Figure 3B,C, the relative 2,7-dichlorofluorescein (DCF) fluorescence intensity of paraquat-exposed nematodes was higher than that of the control, indicating the ROS level was increased by paraquat itself. Interestingly, when the nematodes were pretreated with nostoglycan prior to paraquat exposure, the increased ROS level was significantly reduced (*p* < 0.05), suggesting that reduction of ROS level may contribute to the protective effect of nostoglycan against oxidative stress.

### 2.3. Reduction of Protein Carbonyl and Malonaldehyde Contents and Upregulation of Antioxidant Enzyme Activities by Nostoglycan in C. elegans under Oxidative Stress

One of the impairments caused by excessive ROS is protein carbonyl formation, which leads to rapid degradation of proteins [34]. Another consequence of excessive ROS is the production of malonaldehyde (MDA), a lipid peroxidation product that interacts with DNA and proteins to form toxic adducts [35]. Therefore, we further examined the effect of nostoglycan on the contents of protein carbonyl groups and MDA, both of which can be used as oxidative stress markers. As shown in Table 1, the protein carbonyl and MDA contents were increased after the nematodes were exposed to 10 mM paraquat. When the nematodes were pretreated with 0.5 mg/mL nostoglycan prior to paraquat exposure, however, the elevated protein carbonyl and MDA levels were reduced. These results demonstrate that nostoglycan is capable of inhibiting protein and lipid peroxidation in oxidative stressed animal models, possibly through reducing the ROS level. Interestingly, the MDA content of the nematodes treated with nostoglycan but without paraquat exposure was also significantly lower than that of the unexposed control nematodes, indicating the ability of the polysaccharide per se to inhibit lipid peroxidation. Increasing evidence has shown that lipid peroxidation is implicated in ageing process and age-related diseases, and inhibition of lipid peroxidation can be beneficial to retard ageing and suppress age-related diseases [36,37,38]. For example, *Brassica chinensis* extracts are recently shown to extend the lifespan of *C. elegans*, which is likely related to increased antioxidant enzyme activities and decreased MDA content [37]. Another example is strawberry-rich anthocyanin supplementation, which can ameliorate cardiovascular risk and reduce oxidative stress markers, including serum MDA, in healthy people [38]. Therefore, our results suggest that nostoglycan may have the potential to delay senescence and maintain health. In addition, a number of studies have shown that metal ion chelators can also reduce ROS level and inhibit lipid peroxidation [39,40]. Interestingly, the polysaccharides from several *Nostoc* species, including *N. sphaeroides*, are shown to have strong capacities to chelate metal ions [17,41], suggesting that the antioxidant effect of nostoglycan may also be related with its chelating ability. We have previously shown that a polysaccharide from the medicinal herb *Rubia cordifolia* can activate proteasomal pathway in T-REx293 human embryonic kidney cells expressing amyloid β-peptide [42], which can induce protein and lipid peroxidation in cellular and animal models [43]. Therefore, the cellular protein degradation system, including autophagy and proteasomal pathways, might also contribute to the inhibition of protein and lipid peroxidation by nostoglycan under oxidative stress.

Antioxidant enzymes are important members of cellular antioxidant defense system and play a key role in scavenging excessive ROS and reducing oxidative damages, and many polysaccharides have been shown to regulate the activity of antioxidant enzymes. For example, we have previously shown that the polysaccharide from *Angelica sinensis* can increase superoxide dismutase (SOD) and glutathione peroxidase (GPx) activities in cortical tissue of rats to ameliorate cerebral ischemia injury, a complex pathological process involving oxidative stress [44]. Therefore, we tested whether the antioxidant effect of nostoglycan, including its capacities to improve oxidative survival and reduce ROS level as shown above, was related to its regulation of antioxidant enzyme activities. As shown in Table 1, the SOD activity was increased in the nematodes exposed to 10 mM paraquat alone as compared to the unexposed control, suggesting an adaptive response of the nematodes to oxidative stimulation. This is consistent with previous reports showing that paraquat can stimulate SOD expression and activity in *C. elegans* models [29,45]. However, catalase (CAT) activity of the paraquat-exposed nematodes was reduced as compared to that of the control. Since SOD can convert superoxide anion to hydrogen peroxide and CAT can detoxify hydrogen peroxide to water, these results indicate that paraquat may disrupt the antioxidant defense system and cause accumulation of toxic hydrogen peroxide as demonstrated previously [33]. Interestingly, although the SOD activity of the nematodes treated with 0.5 mg/mL nostoglycan alone was almost unchanged as compared to the control, the SOD activity was further elevated when the nematodes were pretreated with nostoglycan and then exposed to paraquat as compared to that of the paraquat-exposed nematodes. Similarly, we have previously found that *D. indusiata* polysaccharide can increase SOD activity in paraquat-exposed *C. elegans* [29]. As SOD expression and activity can be regulated by many upstream regulators such as the transcription factors DAF-16/FOXO and SKN-1/Nrf2 [45,46], these findings suggest that polysaccharides and paraquat may act differently on SOD expression and activity. For example, paraquat is shown to increase SOD expression through promoting SKN-1 translocation [45] while *D. indusiata* polysaccharide is found to activate DAF-16 but not SKN-1 in *C. elegans* [29]. In addition to SOD, the activity of CAT was also increased in nostoglycan-treated nematodes as compared to the controls with or without paraquat exposure, respectively. The GPx activity was, however, only slightly affected by treatments with nostoglycan, paraquat or both. Taken together, these results suggest that nostoglycan is capable of scavenging toxic superoxide anions and hydroxyl radicals through regulating SOD and CAT activities under oxidative stress and that the upregulation of antioxidant enzymes is likely to play a part in the in vivo antioxidant capacity of the polysaccharide.

### 2.4. Inhibition of Proliferation of Human Tumor Cells by Nostoglycan

**Figure 4:**
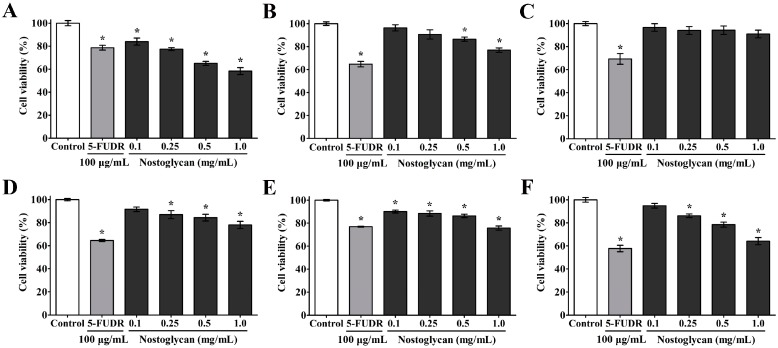
Effect of nostoglycan on the proliferation of tumor cells. Human tumor cell lines A549 (**A**); HepG2 (**B**); HL-60 (**C**); PC3 (**D**); MCF-7 (**E**); and Jurkat (**F**) were used to evaluate the anti-proliferative activity of nostoglycan. The tumor cells were treated with 0.1–1.0 mg/mL nostoglycan or 100 μg/mL 5-FUDR for 48 h, and the cell viabilities were measured by MTT method. All data are normalized to the untreated cells and presented as mean ± SEM of three independent experiments. * *p* < 0.05.

Oxidative stress is closely associated with tumorigenesis and cancer development, e.g. excessive ROS can induce chromosome instability and genetic mutation and also alter gene expression patterns, leading to uncontrolled cell proliferation [12]. On the other hand, antioxidant strategies are considered to be a sensible approach for intervention against cancer progression [47]. Food-derived antioxidants, for example, are known to have potentials to reduce cancer risk and slow cancer development [12,48]. Since nostoglycan has shown antioxidant capacities in several aspects as demonstrated above, we investigated whether the polysaccharide can also inhibit the proliferation of tumor cells using a number of cell lines derived from different human tissues, including lung adenocarcinoma cell line A549, hepatocellular carcinoma cell line HepG2, prostate carcinoma cell line PC3, promyelocytic leukemia cell line HL-60, breast carcinoma cell line MCF-7, and leukemic T cell line Jurkat. The cells were treated with 0.1–1.0 mg/mL nostoglycan for 48 h and then the proliferation rate was determined using 3-(4,5-dimethylthiazol-2-yl)-2,5-diphenyl tetrazolium bromide (MTT) method. As shown in Figure 4, when the concentration of nostoglycan was 1.0 mg/mL, the inhibition against A549, HepG2, PC3, MCF-7, and Jurkat cells was 42%, 24%, 20%, 27% and 36%, respectively, at a comparable inhibition level with 100 μg/mL of 5-fluoro-2′-deoxyuridine (5-FUDR), an anti-metabolite drug that is widely used for cancer therapy [49]. Interestingly, we have previously shown that the extracellular polysaccharide isolated from the spent medium of *N*. *sphaeroides* suspension cultures is also capable of inhibiting the proliferation of several human tumor cell lines, including BGC-823 gastric carcinoma cells and MOLT-4 acute lymphoblastic leukemia cells [50]. Together, these data demonstrate the anti-tumor potential of the antioxidant polysaccharides isolated from *N*. *sphaeroides*.

### 2.5. Induction of Apoptosis through Activation of Capspase-3 in Tumor Cells by Nostoglycan

**Figure 5:**
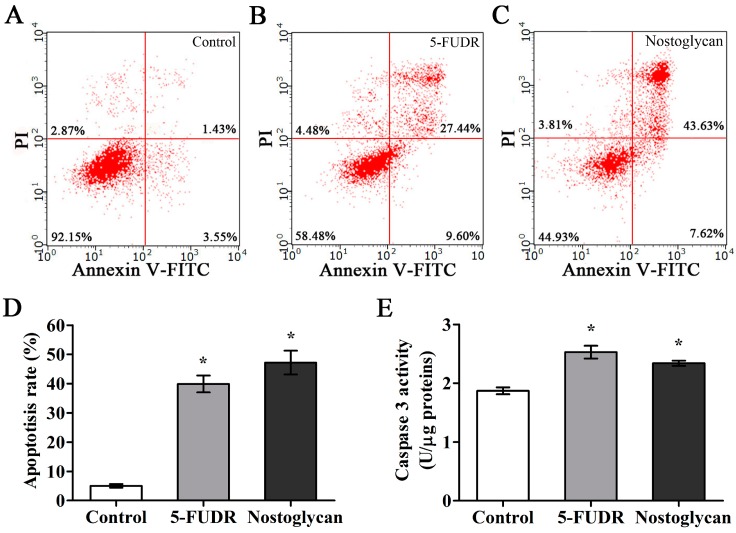
Effect of nostoglycan on apoptosis and caspase-3 activation in tumor cells. Quantitative analysis of apoptosis was performed by flow cytometry using human lung adenocarcinoma cell line A549. The cells were treated with medium (control; **A**), 100 μg/mL 5-FUDR (**B**) or 1.0 mg/mL nostoglycan (**C**) for 48 h and then subjected to Annexin V-FITC/PI staining and flow cytometry analysis. Apoptosis rate was calculated as the percentage of Annexin V-positive cells in >10,000 total cells (**D**). The activity of caspase-3 in A549 cells was determined by colorimetric assay (**E**). Data are presented as mean ± SEM of three independent experiments. * *p* < 0.05.

Apoptosis is a fundamental physiological process of cell death, while loss of apoptotic response contributes significantly to cancer development and multidrug resistance [51]. Targeting cell apoptosis thus offers opportunities to suppress tumor proliferation and improve cancer treatment. Since nostoglycan was capable of inhibiting proliferation of tumor cells, we further tested whether this effect was through modulation of cell apoptosis using the human lung adenocarcinoma cell line A549. The cells were treated with 1.0 mg/mL nostoglycan or 100 μg/mL 5-FUDR for 48 h and then the apoptosis rates were determined by flow cytometry after Annexin V-FITC/propidium iodide (PI) staining. As shown in Figure 5, the apoptosis rates of A549 cells treated with nostoglycan and 5-FUDR were increased to ~47% and ~40%, respectively, while that of untreated cells was ~5%, demonstrating the induction of apoptosis by nostoglycan in A549 cells.

It is well-established that apoptosis occurs mainly through mitochondria- and death receptor-mediated signaling pathways, both of which involve the activation of executor caspases such as caspase-3 [52]. Therefore, to explore the signaling mechanisms of nostoglycan leading to death of tumor cells, we further investigated the effect of nostoglycan on the activation of caspase-3, which plays a central role in the execution phase of cell apoptosis [52]. The A549 cells were treated with 1 mg/mL nostoglycan or 100 μg/mL 5-FUDR for 48 h and then the caspase activity was determined by a colorimetric method. As shown in Figure 5E, the caspase-3 activities were increased in cells treated with either nostoglycan or 5-FUDR, suggesting an involvement of caspase-3 activation in nostoglycan-induced cell apoptosis. In addition to induction of apoptosis, other mechanisms may also be involved in the anti-proliferative effect of *Nostoc* polysaccharides. For example, a polysaccharide from *N. commune* is able to suppress proliferation of MCF-7 cells via modulation of endoplasmic reticulum stress, which is known to trigger unfolded protein response and lead to cell death [53]. Also, a number of studies have shown that the anti-tumor activity of polysaccharides is closely associated with their regulatory effects on immune system [54,55], and, intriguingly, *N. commune* polysaccharide has been previously shown to activate the complement system [2], an important part of the immune system acting to enhance the adaptive immune response. Therefore, other mechanisms are also likely to contribute to the inhibitory effect of nostoglycan and other *Nostoc* polysaccharides on the proliferation of tumor cells.

## 3. Materials and Methods

### 3.1. Preparation of Polysaccharide

Colonies of *Nostoc sphaeroides* Kützing were grown essentially as described [56]. The stock culture was ground and grown in BG11 medium sparged with filtered air in 1 L conical flasks at 20 °C and 80 μE/(m^2^ s) (16 h light and 8 h dark cycle), and the spent medium was regularly exchanged for fresh medium until the diameter of the colonies reached about 0.5 mm. The colonies were then transferred into 5 L glass bottles for a larger scale culture in a similar fashion. Colonies with a diameter of 1–2 mm were collected for outdoor culture in 500 L glass tanks bubbled with filtered air at room temperature with controlled solar irradiance, and the colonies with a diameter of ≥5 mm were harvested and air-dried as *N. sphaeroides* products. Polysaccharide preparation was performed essentially as described previously [4]. In brief, dry *N. sphaeroides* colonies were ground and refluxed in ethanol. After removal of the solvent, the materials were immersed overnight in water and then subjected to hot water extraction. The supernatant was treated with 0.1% papain (pH 6.0) at 40 °C for 2 h and then precipitated with ethanol. The precipitate was redissolved in water, dialyzed, and freeze-dried as *N*. *sphaeroides* polysaccharide (nostoglycan).

### 3.2. Determination of Carbohydrate Content and Analysis of Monosaccharide Composition

Total carbohydrate content was determined by the phenol-sulfuric acid method [57]. Analysis of monosaccharides was performed by gas chromatography as described previously [29]. Briefly, the polysaccharide was first methanolyzed with methanolic HCl using inositol as an internal standard. The resultant methyl glycosides were then subjected to trimethylsilylation and identification on a CP-3800 gas chromatograph (Varian, Palo Alto, CA, USA) with a DB column (30 × 0.25 mm; Agilent, Palo Alto, CA, USA).

### 3.3. FTIR Spectroscopy Analysis

FTIR spectrum of polysaccharide was determined as described previously [58]. Briefly, 1 mg of nostoglycan powder was mixed with 110 mg of KBr powder, and the mixture was pressed into a tablet. The FTIR spectrum was recorded on a Spectrum100 FTIR spectrophotometer (PerkinElmer, Waltham, MA, USA) using 32 scans at a resolution of 4 cm^−1^ in the frequency range of 4000–400 cm^−1^.

### 3.4. Congo Red Binding Assay

The triple helical conformation of polysaccharide was investigated using the azo dye Congo red as described [20]. Briefly, 2 mL of 1 mg/mL nostoglycan solution was mixed with 2 mL of 100 μM Congo red solution, and then 1 mL of NaOH solution with different concentrations was added to achieve a final NaOH concentration of 0–0.5 M. Laminarin and dextran (Sigma, St. Louis, MO, USA) were used as controls for triple helical and random coil conformations, respectively. The λ_max_ of the mixture was recorded by wavelength scanning from 400 to 600 nm using a GENESYS 10S UV-Vis spectrophotometer (Thermo Fisher Scientific, Waltham, MA, USA).

### 3.5. Surface Morphology Analysis

The surface morphology of nostoglycan was analyzed using SEM as described previously [59]. Briefly, the dried nostoglycan powder was mounted on a copper stub using a double-sided adhesive carbon tape, and sputtered with a layer of gold using a sputter coater. The sample was then observed by a Hitachi S-3700N SEM (Hitachi, Tokyo, Japan) at an acceleration voltage of 15 kV.

### 3.6. Assessment of Moisture Absorption and Retention

The moisture absorption and retention capacities of polysaccharides were evaluated using moisture weight gain-and-loss assay as described previously [4]. To determine the *R*a, the dried nostoglycan and chitosan (as a control) were placed in a sealed humidity chamber with 43% or 81% RH for moisture absorption and their *R*a was calculated according to the weight gained after an indicated time. To determine the *R*r, the dried polysaccharides were first fully humidified in a sealed chamber containing water and then transferred to the 43% RH chamber to dehumidify for the indicated times. After that, the polysaccharides were transferred to a desiccation chamber containing dried silica gel to further dehydrate for the indicated times. The *R*r of polysaccharides was calculated according to the weight loss of the moisturized polysaccharides after dehydration for an indicated time.

### 3.7. Determination of Viscosity

The relative viscosity of nostoglycan was determined using an Ubbelohde capillary viscometer (ShenLi Glass Labware, Shanghai, China) as previously described [60]. Briefly, 20 mg of nostoglycan was dissolved in 20 mL of water and passed through a 0.45 μm Millipore membrane filter. Then 10 mL of the nostoglycan solution or water was transferred into the viscometer, and the efflux time of nostoglycan solution and water was measured respectively. The relative viscosity was calculated as the ratio of the efflux time of the polysaccharide solution to that of water. Apple pectin and agar (Sigma) were used for comparison.

### 3.8. Nematode Maintenance

Both *Caenorhabditis elegans* strain N2 (wild type) and *Escherichia coli* strain OP50 were obtained from the *Caenorhabditis* Genetics Center (University of Minnesota, Minneapolis, MN, USA). *C. elegans* was maintained at 20 °C on nematode growth media agar plates with *E*. *coli* OP50 as food. Synchronization of nematodes was performed using the standard alkaline hypochlorite method.

### 3.9. Paraquat Survival Assay in C. elegans

The paraquat survival assay was performed using *C. elegans* in liquid culture as described previously [30]. In brief, synchronized L1 larvae were grown in S medium until L4 and then 5-FUDR (Sigma) was added to suppress reproduction. After further incubation for 24 h, the young adult nematodes were treated with 0.1–0.5 mg/mL of nostoglycan for 48 h and then exposed to 70 mM paraquat. The numbers of live and dead nematodes were scored every 12 h based on their movement until all dead. EGCG (final concentration of 100 μM) was used as a positive control.

### 3.10. Determination of ROS Level in C. elegans

The ROS level in *C. elegans* was determined using the fluorescent probe DCFH-DA as previously described [30]. Briefly, synchronized L1 nematodes were pretreated with 0.5 mg/mL of nostoglycan for 48 h and then exposed to 10 mM paraquat for 24 h. After wash with M9 buffer, the nematodes were dispensed into black 96-well plates (90 nematodes/well; 900 nematodes for each treatment) by a COPAS Biosort instrument (Union Biometrica, Inc., Holliston, MA, USA). DCFH-DA was then added at a final concentration of 50 μM and the plates were incubated at 20 °C for 14 h. The images of fluorescence from the resulting DCF were captured by ImageXpress Micro System (Molecular Devices, Sunnyvale, CA, USA). The fluorescence intensity was determined using a Fluoroskan Ascent FL microplate reader (Thermo) at an excitation of 485 nm and an emission of 535 nm.

### 3.11. Determination of Protein Carbonyl and MDA Contents and Antioxidant Enzyme Activities in C. elegans

Protein carbonyl content [61], MDA content [62], and antioxidant enzyme activities [62] were determined as described previously. Briefly, synchronized L1 nematodes were pretreated with 0.5 mg/mL of nostoglycan and then exposed to 10 mM paraquat as above. Approximately 3000 nematodes were homogenized in 400 μL of Western and IP lysis buffer (Beyotime, Shanghai, China). The lysate was collected by centrifugation and used for the following determinations using commercial assay kits, respectively: protein carbonyl content (Jiancheng, Nanjing, China); MDA content and SOD, CAT and GPx activities (Beyotime); and protein content (Thermo), which was used to normalize protein carbonyl and MDA contents and antioxidant enzyme activities.

### 3.12. Tumor Cell Lines

The human tumor cell lines—including A549, HepG2, PC3, HL-60, MCF-7, and Jurkat—were obtained from the Cell Resource Center of Shanghai Institutes for Biological Sciences, the Chinese Academy of Sciences (Shanghai, China). The cells were maintained in RPMI-1640 medium (A549, HepG2, HL-60, and Jurkat), DMEM/F-12 medium (PC3), or DMEM medium with high glucose (MCF-7). The culture medium was supplemented with 10% fetal bovine serum, 100 U/mL penicillin, and 100 μg/mL streptomycin. The cells were grown at 37 °C in a humidified atmosphere with 5% CO_2_. For subculture of the adherent cell lines A549, HepG2, PC3, and MCF-7, the cells were passaged by trypsinization when the confluence reached about 80%. For maintenance of the suspension cell lines HL-60 and Jurkat, the cells were diluted with fresh medium to a density of 1 × 10^5^ cells/mL when the cell density reached 1 × 10^6^ cells/mL.

### 3.13. Cell Viability Assay

Cell viability was assessed using MTT as described previously [50]. Briefly, 100 μL of cells were seeded at a density of 1 × 10^4^ cells/mL in clear 96-well flat-bottom polystyrene microplates (NEST, Wuxi, China), which were tissue-culture treated and sterilized. After growing in normal medium for 24 h, the medium was removed and the cells were treated with a given concentration of nostoglycan (0.1, 0.25, 0.5, and 1.0 mg/mL) in respective serum-free medium for another 48 h. Then 10 μL of 5 mg/mL MTT was added to each well and the plates were incubated further at 37 °C for 4 h. After removal of the medium, 100 μL of DMSO was added to dissolve the formazan crystals. The absorbance was measured at 570 nm on a microplate reader (Thermo Fisher, Waltham, MA, USA). 5-FUDR (100 μg/mL, final concentration) was used as a positive control. All data were normalized to the absorbance of the control without polysaccharide or 5-FUDR treatment.

### 3.14. Flow Cytometry Analysis of Cell Apoptosis

Apoptosis of human lung adenocarcinoma A549 cells was analyzed by flow cytometry using Annexin V/PI staining as described [63]. Briefly, 2 mL of the cells were seeded at a density of 1 × 10^5^ cells/mL in clear 6-well flat-bottom polystyrene plates (NEST, Wuxi, China) and incubated for 24 h. After treatment with 1.0 mg/mL nostoglycan or 100 μg/mL 5-FUDR (as a control) for another 48 h, the cells were incubated with Annexin V and PI according to the instructions of Apoptosis Detection Kit (KeyGen, Nanjing, China) and then analyzed using a flow cytometer (Merck Millipore, Darmstadt, HE, Germany) with an excitation of 488 nm and an emission of 530 nm. Apoptosis was expressed as percentage of Annexin V-positive cells in >10,000 total cells analyzed in the gated region.

### 3.15. Measurement of Caspase-3 Activity

The caspase-3 activity was measured using a caspase colorimetric kit (Beyotime) as described [64]. Briefly, A549 cells were treated with nostoglycan or 5-FUDR as described in the cell apoptosis analysis. The cells were collected, washed with PBS, and lysed in the cell lysis buffer provided in the kit. The supernatant was collected and mixed with the reaction buffer in 96-well plates. After addition of the caspase-3 colorimetric substrate Ac-DEVD-ρNA, the plates were incubated at 37 °C for 4 h and read at 405 nm in the microplate reader (Thermo Fisher). Data were normalized to protein content.

### 3.16. Statistical Analysis

Statistical analysis was performed primarily by GraphPad Prism 5.0 for Windows (GraphPad Software, San Diego, CA, USA). Statistical significance was determined by one-way ANOVA followed by Tukey’s post hoc test. *C. elegans* survival data were analyzed by Kaplan–Meier method and log-rank test using SPSS 17.0 for Windows (SPSS, Chicago, IL, USA). All experiments were performed at least three times. A probability value of *p* < 0.05 was considered statistically significant.

## 4. Conclusions

In this study, we first investigated the physicochemical properties of nostoglycan, a polysaccharide isolated from cultured *N. sphaeroides* colonies, and found that the polysaccharide has strong moisture absorption and retention capabilities and a high relative viscosity. Then we demonstrate that nostoglycan can increase survival rate, reduce ROS levels and protein carbonyl and MDA contents, and enhance SOD and CAT activities of *C. elegans* under increased oxidative stress induced by paraquat. We also show that nostoglycan is able to inhibit the proliferation of several different types of tumor cells. Using the human lung adenocarcinoma cell line A549, we further reveal that nostoglycan can induce apoptosis of tumor cells via caspase-dependent pathway. These results provide an important insight into the potentials of nostoglycan in food and health industries.

## Figures and Tables

**Table 1:** Effect of nostoglycan on protein carbonyl and malondialdehyde contents and antioxidant enzyme activities in *C. elegans*.

Treatment	Protein Carbonyl Content ^a^	MDA Content ^b^	SOD Activity ^c^	CAT Activity ^d^	GPx Activity ^c^
Control	0.88 ± 0.15	7.60 ± 0.54	49.02 ± 4.66	1.20 ± 0.02	15.59 ± 1.63
Nostoglycan	0.82 ± 0.12	4.94 ± 0.31 ^e^	46.81 ± 6.84	1.32 ± 0.02 ^e^	16.87 ± 1.50
Paraquat	1.71 ± 0.14 ^e^	10.39 ± 0.87 ^e^	69.97 ± 3.51 ^e^	1.08 ± 0.02 ^e^	15.09 ± 0.76
Nostoglycan + Paraquat	1.02 ± 0.18 ^f^	7.28 ± 0.46 ^f^	97.88 ± 6.72 ^f^	1.21 ± 0.02 ^f^	14.19 ± 1.48

The nematodes were treated with or without 0.5 mg/mL of nostoglycan followed by exposure to 10 mM of paraquat. ^a^ Protein carbonyl content, nmol/mg proteins; ^b^ MDA content, μM/mg proteins; ^c^ SOD and GPx activities, U/mg proteins; ^d^ CAT activity, U/μg proteins; ^e^ *p* < 0.05, compared with the control nematodes; ^f^ *p* < 0.05, compared with paraquat-intoxicated nematodes.
